# Acoustically propelled nano- and microcones: fast forward and backward motion

**DOI:** 10.1039/d1na00655j

**Published:** 2021-10-26

**Authors:** Johannes Voß, Raphael Wittkowski

**Affiliations:** Institut für Theoretische Physik, Center for Soft Nanoscience, Westfälische Wilhelms-Universität Münster D-48149 Münster Germany raphael.wittkowski@uni-muenster.de

## Abstract

We focus on cone-shaped nano- and microparticles, which have recently been found to show particularly strong propulsion when they are exposed to a traveling ultrasound wave, and study based on direct acoustofluidic computer simulations how their propulsion depends on the cones' aspect ratio. The simulations reveal that the propulsion velocity and even its sign are very sensitive to the aspect ratio, where short particles move forward whereas elongated particles move backward. Furthermore, we identify a cone shape that allows for a particularly large propulsion speed. Our results contribute to the understanding of the propulsion of ultrasound-propelled colloidal particles, suggest a method for separation and sorting of nano- and microcones concerning their aspect ratio, and provide useful guidance for future experiments and applications.

## Abstract 2

We focus on cone-shaped nano- and microparticles, which have recently been found to show strong propulsion when exposed to a traveling ultrasound wave, and study by computer simulations how their propulsion depends on the cones' aspect ratio.

## Graphical abstract

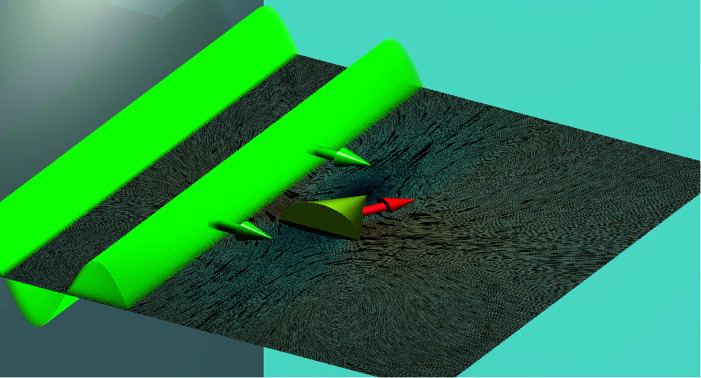

## I. Introduction

Research on active nano- and microparticles is strongly motivated by their complex nonequilibrium behavior, which is interesting from a fundamental point of view, and by the wide range of possible future applications of such particles.^1–4^ Among the most important potential fields of application are medicine^5–12^ and materials science.^13–19^ However, most of the active particles that have been developed so far are mainly useful for fundamental research but not suitable for applications, *e.g.*, since their propulsion runs quickly out of fuel, requires a very particular chemical environment, or is not biocompatible.^9,20–25^ A realization of active particles that is particularly useful for applications is acoustically propelled particles.^26–60^ Some of their advantages are that the particles can easily and permanently be supplied with energy *via* ultrasound, that the propulsion mechanism works in various types of fluids and soft materials, and that it is biocompatible.

There exist two different types of acoustically propelled particles: rigid particles^26–28,31–34,37,38,40,41,43,44,46,51,55–57,60–65^ and particles with movable components.^23,40,45,50,53,54,58,59,66^ The rigid particles are easier to produce in large numbers and thus of special relevance with respect to future applications, where usually a large number of particles is required. There exist also some hybrid particles that combine acoustic propulsion with other propulsion mechanisms.^57,62,64,65,67,68^

In recent years, ultrasound-propelled nano- and microparticles have been intensively investigated.^26–28,30,32,34,37–40,43–46,51–55,57–61,63–65,69–71^ Besides two articles that are based on analytical approaches^61,63^ and an article that relies on direct computational fluid dynamics simulations,^51^ a large number of experimental studies have been published so far.^26–28,30,32,38–40,44,45,49,50,53–55,57,60,64,65,69^ It was found that the propulsion of the particles is strongly linked with an asymmetry of their shape.^61,63^ Also, an anisotropic mass density of the particle was found to affect its motion.^63^ In the previous work, mostly cylindrical particles with a concave end and a convex end were studied.^26–28,30,32–34,39,45,54,55,60,65^ As a limiting case, which corresponds to a very short cylindrical particle with concave and convex ends, also half-sphere cups (nanoshells) were considered.^38,51,64^ Recently, half-sphere-shaped particles, cone-shaped particles, and spherical-as well as conical-cup-like particles were compared with respect to their propulsion.^51^ In two other studies, gear-shaped microspinners^44,69^ were addressed, and there are a few additional publications that focus on particles with movable components.^23,40,50,53,66^

Among the rigid particles with mainly translational motion that have been addressed so far, cone-shaped particles and conical-cup-shaped particles showed the fastest propulsion, where the speeds of cone-shaped and conical-cup-shaped particles differed only slightly.^51^ Since cone-shaped particles have a simpler shape, which facilitates their fabrication, and a larger volume, which is advantageous for delivery of drugs or other substances, than conical-cup-shaped particles, the former particles have been identified as particularly suitable candidates for efficient ultrasound-propelled particles that could be used in future experiments and applications. Cone-shaped particles can be produced, *e.g.*, by electrodeposition,^42,72^ or directly be found, *e.g.*, in the form of carbon nanocones,^73^ in large numbers. Up to now, however, only ultrasound-propelled cone-shaped particles with a particular aspect ratio have been studied,^51^ although the aspect ratio can have a strong influence on the efficiency of the particles' propulsion.^63^ Given that previous studies found for the short spherical-cup-shaped particles motion towards the particles' convex end^38,51,64^ but for the longer cylindrical particles with concave and convex ends motion towards the concave end,^39^ also the direction of propulsion can depend on the aspect ratio.

Therefore, in this article we investigate the acoustic propulsion of cone-shaped nano- and microparticles with a constant mass density in more detail. Using direct acoustofluidic simulations, we study how the propulsion of acoustically propelled nano- and microcones depends on their aspect ratio and we determine an aspect ratio that is associated with particularly fast and thus efficient propulsion. In contrast to all but one^40^ previous experimental studies, we assume that the particles are exposed to a traveling ultrasound wave since this scenario is more realistic for future applications of the particles than standing ultrasound waves that are usually considered.

## II. Methods

This work is based on a similar setup and procedure as ref. 51 since the methodology used there has been proven to be very suitable for studying ultrasound-propelled particles. We consider a particle that is surrounded by water and exposed to ultrasound. Using direct acoustofluidic simulations, where the compressible Navier–Stokes equations are solved numerically, the propagation of ultrasound through the water and the interaction with the particle are calculated. These calculations allow to determine the sound-induced forces and torques acting on the particle, from which in turn we calculate the particle's translational and angular propulsion velocities.

**Fig. 1:**
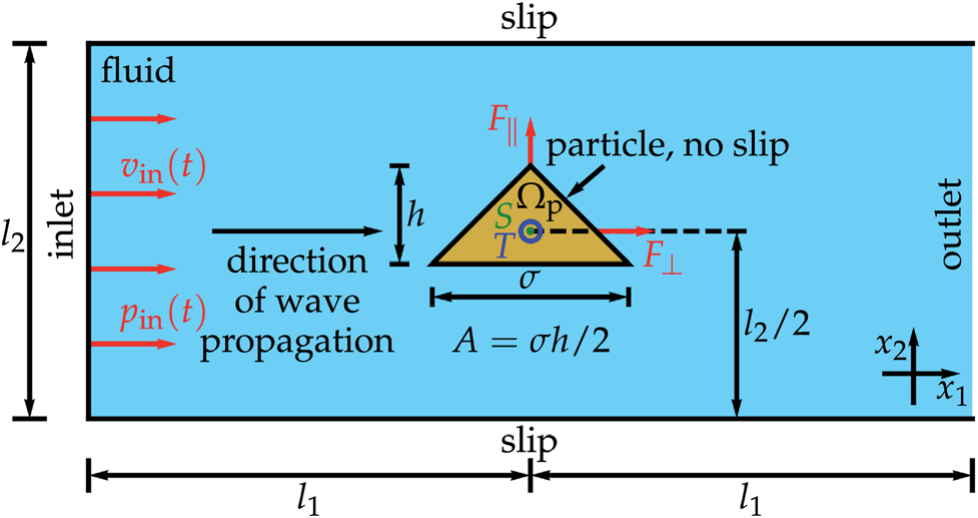
The considered setup. A rigid cone-shaped particle is in the middle of a fluid-filled rectangular domain. The particle has width *σ*, height *h*, and a fixed cross-section area *A*, and is described by a particle domain *Ω*_p_. Furthermore, the rectangular domain has width 2*l*_1_ and height *l*_2_ and the center of mass S of the particle is in the middle of the rectangular domain. At the inlet, a traveling ultrasound wave is entering the fluid-filled domain. For this purpose, an inflow velocity *v*_in_(*t*) and pressure *p*_in_(*t*) are prescribed at the inlet. The ultrasound wave propagates through the fluid, where slip boundary conditions are used for the lateral boundaries of the rectangular domain. At the particle, for which no-slip boundary conditions are used, the ultrasound exerts a propulsion force with time-averaged components *F*_∥_ and *F*_⊥_ parallel and perpendicular to the particle's orientation, respectively, as well as a time-averaged torque *T*. When the ultrasound wave reaches the end of the domain, it leaves the domain through the outlet.

Fig. 1 shows the setup in detail. We consider a particle with a conical shape in two spatial dimensions. The particle is oriented perpendicular to the direction of wave propagation, has a fixed cross-section area *A*, and is described by a particle domain *Ω*_p_. Its aspect ratio *χ* = *h*/*σ* with the particle's height *h* and diameter *σ* is varied. The position of the particle is fixed. This means that the results of the simulations are valid for a particle which is held in place. Such a particle can be seen as a free moving particle in the limiting case of an infinite mass density. This limiting case can, in turn, be considered as an upper bound for a free moving particle made of a material with a high mass density like gold, which is a widely used material for such particles.^26,30,32,34,37–39,42–44,46,48,55^

The particle is positioned in the middle of a water-filled rectangular domain so that the center of mass of the rectangle and the center of mass S of the particle coincide. One edge of the rectangular domain has length *l*_2_ = 200 μm and is perpendicular to the direction of ultrasound propagation. The other edge is parallel to the direction of ultrasound propagation and has length 2*l*_1_. We choose a Cartesian coordinate system such that the *x*_1_ axis is parallel to the direction of ultrasound propagation and the *x*_2_ axis is perpendicular to that direction, *i.e.*, the coordinate axes are parallel to the edges of the rectangular domain. The ultrasound wave has frequency *f* = 1 MHz and enters the rectangular domain at the edge perpendicular to the sound-propagation direction. We prescribe the incoming ultrasound wave by a time-dependent inflow pressure *p*_in_(*t*) = Δ*p* sin(2π*ft*) and velocity *v*_in_(*t*) = Δ*v*  sin(2π*ft*) perpendicular to the inlet with the pressure amplitude Δ*p* = 10 kPa, which is much lower than the normal pressure *p*_0_ = 101 325 Pa and consistent with previous work,^51^ and the velocity amplitude Δ*v* = Δ*p*/(*ρ*_0_*c*_f_) = 6.75 mm s^−1^. Here, *ρ*_0_ = 998 kg m^−3^ is the mass density of the initially quiescent fluid, and *c*_f_ = 1484 m s^−1^ is the sound velocity in the fluid. The acoustic energy density of this wave is *E* = Δ*p*^2^/(2*ρ*_0_*c*_f_^2^) = 0.0227 J m^−3^. Both Δ*v* and *E* are not prescribed directly but follow from the value chosen for the pressure amplitude Δ*p*. Note that energy densities below *E*_max_ = 4.9 J m^−3^ are considered as harmless in medical applications.^74^ In experiments reported in the literature,^39,40,55^ mostly a propulsion voltage of 10 V was applied. A voltage of ≲10 V can be assumed to correspond to an energy density of 10–100 J m^−3^.^75^ Thus, in the experiments from the literature, the energy density was much larger than the energy density used in the present work and even much larger than the maximum harmless energy density *E*_max_. After a distance *l*_1_ = *λ*/4, where *λ* is the wavelength of the ultrasound wave *λ* = 1.484 mm, the wave reaches the fixed rigid particle. The interaction of the ultrasound with the particle leads to time-averaged forces *F*_∥_ parallel and *F*_⊥_ perpendicular to the particle orientation as well as to a time-averaged torque *T* relative to the reference point S acting on the particle. After a further distance *l*_1_, the wave leaves the domain through an outlet at the edge of the water domain opposing the inlet. The full boundary of the particle is described by a no-slip condition, as it is common for treating solid–liquid interfaces in fluid dynamics, and at the edges of the water domain parallel to the direction of sound propagation we assume a slip condition to minimize the effect of these boundaries on the propagation of the ultrasound wave.

In the simulations, we numerically solve the continuity equation for the mass-density field of the fluid, the compressible Navier–Stokes equation, and a linear constitutive equation for the fluid's pressure field. Thus we are avoiding approximations like perturbation expansions that are used in most previous studies using analytical^61,63^ or numerical^40,44,64^ methods. For solving these equations, we used the finite volume method implemented in the software package OpenFOAM.^76^ We applied a structured mixed rectangular–triangular mesh with about 300 000 cells, where the cell size Δ*x* is very small close to the particle, and larger far away from it. Concerning the time integration, an adaptive time-step method is used with a time-step size Δ*t* such that the Courant–Friedrichs–Lewy number1
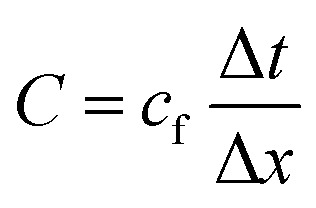
is smaller than one. To get sufficiently close to the stationary state, we simulated a time interval with a duration of *t*_max_ = 500*τ* or more, where *τ* is the period of the ultrasound wave. An individual simulation required a computational expense of typically 36 000 CPU core hours. The reason for this expense is the necessary fine discretization in space and time relative to the large spatial and temporal domains.

Through the simulations, we calculated the time-dependent force and torque acting on the particle in the laboratory frame. Since the particle has no-slip boundary conditions and is fixed in space, the fluid velocity is zero at the fluid–particle interface. So the force and torque can be calculated by the integral of the stress tensor Σ over the particle surface. The force *F⃑*^(*p*)^ + *F⃑*^(*v*)^ and torque *T*^(*p*)^ + *T*^(*v*)^ consist of two components, namely a pressure component (superscript “(*p*)”) and a viscosity component (superscript “(*v*)”) with^77^2
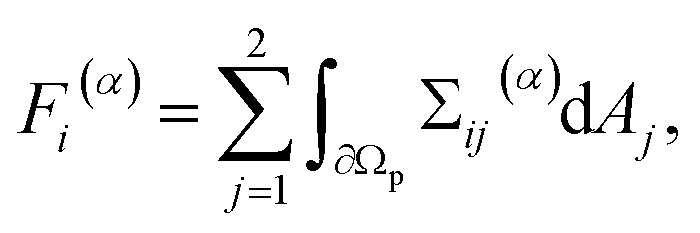
3
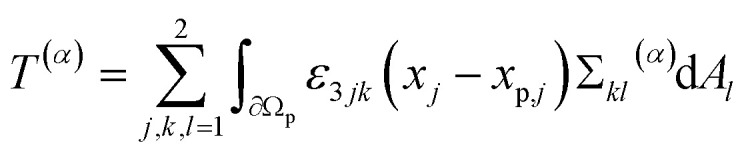
for *α* ∈ {*p*, *v*}. Here, Σ^(*p*)^ and Σ^(*v*)^ are the pressure component and the viscous component of the stress tensor, respectively. d*A⃑*(*x⃑*) = (d*A*_1_(*x⃑*),d*A*_2_(*x⃑*))^T^ is the normal and outwards oriented surface element of ∂*Ω*_p_ at position *x⃑* when *x⃑* ∈ ∂*Ω*_p_, *ε*_*ijk*_ the Levi-Civita symbol, and *x⃑*_p_ the position of S. To obtain the time-averaged stationary values, we locally averaged over one period and extrapolated towards *t* → *∞* using the extrapolation procedure described in ref. 51.

With this procedure, we get the force *F⃑* = *F⃑*_*p*_ + *F⃑*_*v*_ with pressure component *F⃑*_*p*_ = 〈*F⃑*^(*p*)^〉 and viscous component *F⃑*_*v*_ = 〈*F⃑*^(*v*)^〉 as well as the torque *T* = *T*_*p*_ + *T*_*v*_ with components *T*_*p*_ = 〈*T*^(*p*)^〉 and *T*_*v*_ = 〈*T*^(*v*)^〉 acting on the particle, where 〈⋅〉 denotes the time average. To calculate the translational–angular velocity vector 
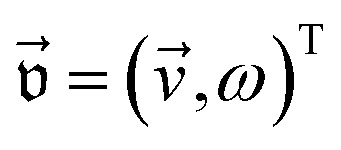
 with the particle's translational velocity 
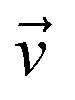
 and angular velocity *ω*, we define the force-torque vector 
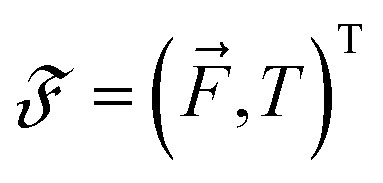
. Then the values of 
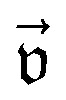
 can be calculated with the Stokes law as^78^4
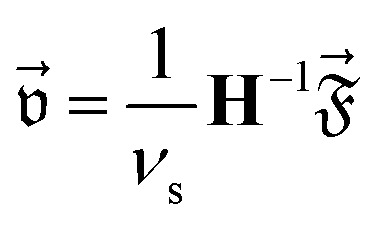
with the fluid's shear viscosity *ν*_s_ and the hydrodynamic resistance matrix5
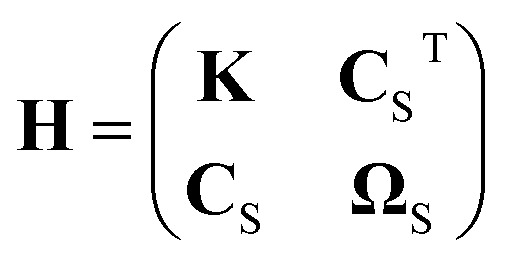
Here, **K**_S_, **C**_S_, and **Ω**_S_ are 3 × 3-dimensional submatrices and the subscript S denotes the particle's center of mass as the reference point.

Since we consider a system with two spatial dimensions to keep the computational effort for the simulations manageable, we cannot use eqn (2)–(4) directly. Hence, we assign a thickness of 1 μm to the particle, so that **H** can be calculated.^79,80^ The general structure of **H** for a particle with a shape as we study here is6
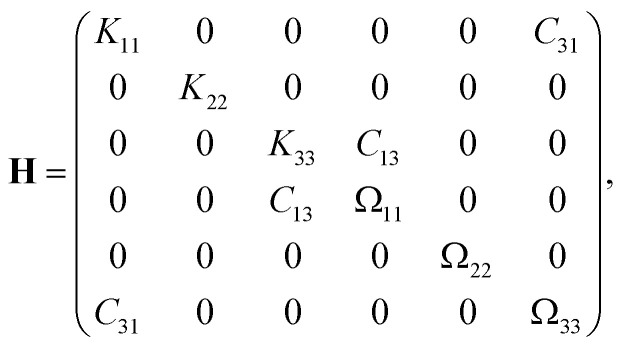
where the values of the nonzero elements are given in Table 1 for each aspect ratio considered in this work. By neglecting the contributions *K*_33_, *C*_13_, *Ω*_11_, and *Ω*_22_ that correspond to the lower and upper surfaces of the particle, we can then use the three-dimensional versions of eqn (2)–(4). From the hydrodynamic resistance matrix **H**, we can also calculate the diffusion tensor.7
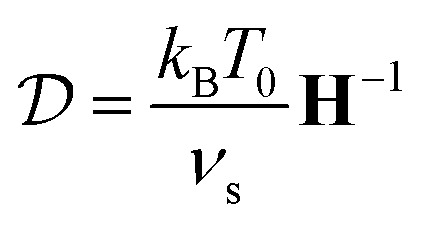
of a particle, where *k*_B_ is the Boltzmann constant and *T*_0_ the temperature of the fluid.

**Table 1:** Elements of the hydrodynamic resistance matrix **H** for a particle with a triangular cross section as shown in Fig. 1, a thickness of 1 μm in the third dimension, and the center of mass as the reference point for different aspect ratios *χ* = *h*/*σ* with the particle's height *h* and diameter *σ*

*χ*	*K*_11_/μm	*K*_22_/μm	*K*_33_/μm	*C*_13_/μm^2^	*C*_31_/μm^2^	*Ω*_11_/μm^3^	*Ω*_22_/μm^3^	*Ω*_33_/μm^3^
0.25	8.58	11.29	8.98	−0.38	0.61	3.44	4.76	4.75
0.5	8.49	9.72	8.14	−0.14	0.4	3	3.36	2.63
0.75	8.76	9.07	7.94	−0.03	0.11	3.06	3.03	2.22
1	9.03	8.77	7.94	−0.01	−0.14	3.18	2.96	2.27
1.5	9.69	8.57	8.13	0.25	−0.54	3.68	2.97	2.68
2	10.15	8.55	8.39	0.43	−0.9	4.25	3.08	3.29
2.5	10.61	8.56	8.59	0.6	−1.21	4.86	3.17	3.97
3	11.02	8.64	8.84	0.77	−1.5	5.53	3.29	4.69
3.5	11.41	8.75	9.08	0.95	−1.79	6.23	3.41	5.47
4	11.67	8.87	9.33	1.1	−2.07	6.94	3.51	6.27

We determine the components of *F⃑* and 
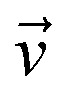
 parallel and perpendicular to the particle's orientation, *i.e.*, parallel to the *x*_2_ and *x*_1_ axes, respectively. These components are the parallel force *F*_∥_ = (*F⃑*)_2_ = *F*_∥,*p*_ + *F*_∥,ν_, with its pressure component *F*_∥,*p*_ = (〈*F⃑*^(*p*)^〉)_2_ and viscous component *F*_∥,ν_ = (〈*F⃑*^(ν)^〉)_2_, perpendicular force *F*_⊥_ = (〈*F⃑*_⊥_〉)_1_ = *F*_⊥,*p*_ + *F*_⊥,ν_ with the components *F*_⊥,*p*_ = (〈*F⃑*^(*p*)^〉)_1_ and *F*_⊥,ν_ = (〈*F⃑*^(ν)^〉)_1_, parallel speed 
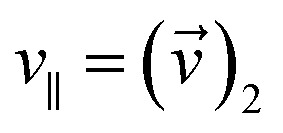
, and perpendicular speed 
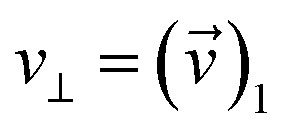
.

Nondimensionalization of the governing equations leads to four dimensionless numbers: the Euler number Eu corresponding to the pressure amplitude of the ultrasound wave entering the simulated system, the Helmholtz number He corresponding to the frequency of the ultrasound wave, a Reynolds number Re_b_ corresponding to the bulk viscosity, and another Reynolds number Re_s_ corresponding to the shear viscosity. Table 2 shows the names and symbols of the parameters that are relevant for our simulations and their values that we have chosen in analogy to the values used in ref. 51. By using the parameter values from Table 2, the dimensionless numbers for our simulations have the following values:

8 Eu = Δ*p*/(*ρ*_0_Δ*v*^2^) ≈ 219 919,

9
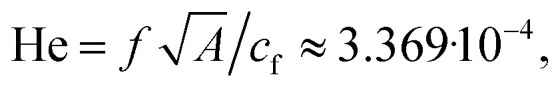
10
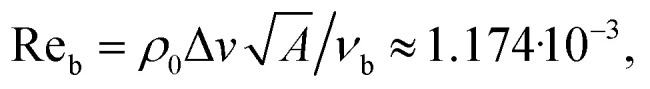
11
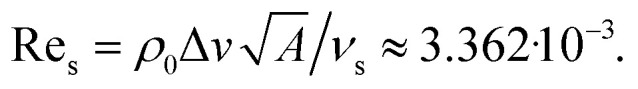


**Table 2:** Parameters that are relevant for our simulations and their values, which are chosen similar to those in ref. 51. The values of the speed of sound *c*_f_, mean mass density *ρ*_0_, shear viscosity *ν*_s_, and bulk viscosity *ν*_b_ are calculated for water at rest at normal temperature *T*_0_ and normal pressure *p*_0_

Name	Symbol	Value
Particle cross-section area	*A*	0.25 μm^2^
Particle diameter-height ratio	*χ* = *h*/*σ*	0.25–4
Particle diameter	*σ*	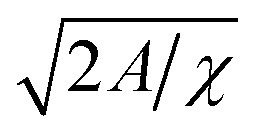
Particle height	*h*	*σχ*
Sound frequency	*f*	1 MHz
Speed of sound	*c*_f_	1484 m s^−1^
Time period of sound	*τ* = 1/*f*	1 μs
Wavelength of sound	*λ* = *c*_f_/*f*	1.484 mm
Temperature of fluid	*T*_0_	293.15 K
Mean mass density of fluid	*ρ*_0_	998 kg m^−3^
Mean pressure of fluid	*p*_0_	101 325 Pa
Initial velocity of fluid	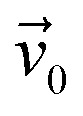	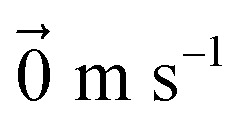
Sound pressure amplitude	Δ*p*	10 kPa
Flow velocity amplitude	Δ*v* = Δ*p*/(*ρ*_0_*c*_f_)	6.75 mm s^−1^
Acoustic energy density	*E* = Δ*p*^2^/(2*ρ*_0_*c*_f_^2^)	22.7 mJ m^−3^
Shear/dynamic viscosity of fluid	*ν*_s_	1.002 mPa s
Bulk/volume viscosity of fluid	*ν*_b_	2.87 mPa s
Inlet-particle or particle-outlet distance	*l*_1_	*λ*/4
Inlet length	*l*_2_	200 μm
Mesh-cell size	Δ*x*	15 nm to 1 μm
Time-step size	Δ*t*	1–10 ps
Simulation duration	*t*_max_	500*τ*
Euler number	Eu	219 919
Helmholtz number	He	3.369·10^−4^
Bulk Reynolds number	Re_b_	1.174·10^−3^
Shear Reynolds number	Re_s_	3.362·10^−3^
Particle Reynolds number	Re_p_	<6·10^−8^

The Euler number Eu denotes the ratio of the pressure amplitude and the inertial force density of the liquid that corresponds to the oscillatory motion of the liquid resulting from the ultrasound wave. Since Eu ≫ 1, the inertial force density is negligible compared to the pressure amplitude. With the Helmholtz number He, the ratio of the size of the particle and the wavelength of the ultrasound is denoted. Here, He ≪ 1 since the particle is much smaller than the wavelength. The Reynolds numbers correspond to the ratio of the inertial forces and viscous forces acting on the particle. Since Re_b_ ≪ 1 and Re_s_ ≪ 1, the viscous forces clearly dominate. Note that the Reynolds number12
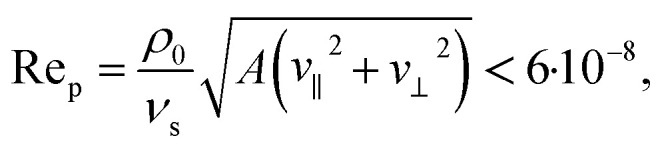
which characterizes the particle motion through the fluid, is also close to zero. Therefore, no turbulence and thus no dissipation by associated vortices are to be expected in the studied system. (This, however, does not exclude the formation of vortices for other reasons.)

## III. Results and discussion

**Fig. 2:**
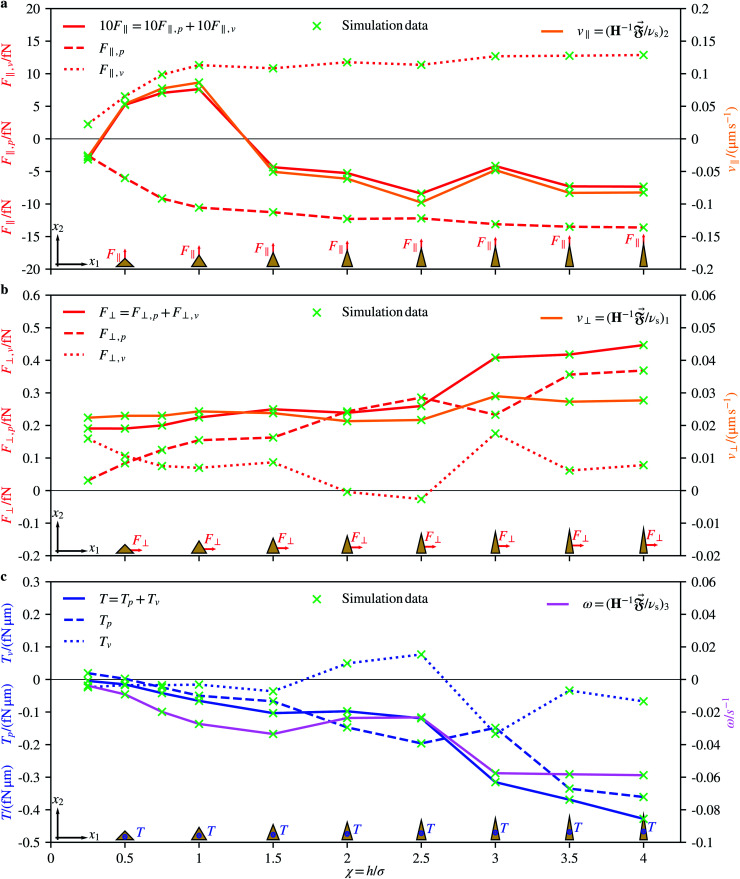
(a) Simulation data for the forces *F*_∥,*p*_ and *F*_∥,*v*_ acting on a particle with triangular cross section and aspect ratio *χ* parallel to its orientation, their sum *F*_∥_ = *F*_∥,*p*_ + *F*_∥,*v*_, and the corresponding propulsion velocity *v*_∥_ for various values of *χ*. (b) The corresponding forces *F*_⊥,*p*_ and *F*_⊥,*v*_ for the direction perpendicular to the particle orientation, their sum *F*_⊥_ = *F*_⊥,*p*_ + *F*_⊥,*v*_, and the velocity *v*_⊥_ for different values of *χ*. (c) Results of the simulation for the torque components *T*_*p*_ and *T*_*v*_ acting on the particle, their sum *T* = *T*_*p*_ + *T*_*v*_, and the corresponding angular velocity *ω* for different values of *χ*.

Fig. 2 shows our results for the propulsion-force components *F*_∥_ and *F*_⊥_, propulsion torque *T*, translational-propulsion-velocity components *v*_∥_ and *v*_⊥_, and angular propulsion velocity *ω* as functions of the aspect ratio *χ* ∈ [0.25,4].

The force *F*_∥_ and velocity *v*_∥_ have a strong dependence on the aspect ratio *χ*. This includes even a sign change. Both curves have qualitatively the same course. They start at *χ* = 0.25 with negative propulsion force *F*_∥_ = −0.32 fN and velocity *v*_∥_ = −0.028 μm s^−1^. Increasing *χ* leads to a positive sign of *F*_∥_ and *v*_∥_ until about *χ* = 1 where the force is maximal with *F*_∥_ = 0.76 fN and also the speed reaches its maximum *v*_∥_ = 0.086 μm s^−1^. Afterwards, the propulsion force and velocity decrease to and remain at negative values. The globally maximal amplitude is reached at *χ* = 2.5, where *F*_∥_ = −0.84 fN and *v*_∥_ = −0.098 μm s^−1^. In the further course of the curves, the values rise until *χ* = 3 and then decrease again until *χ* = 4, where the values saturate at *F*_∥_ = −0.73 fN and *v*_∥_ = −0.082 μm s^−1^.

**Fig. 3:**
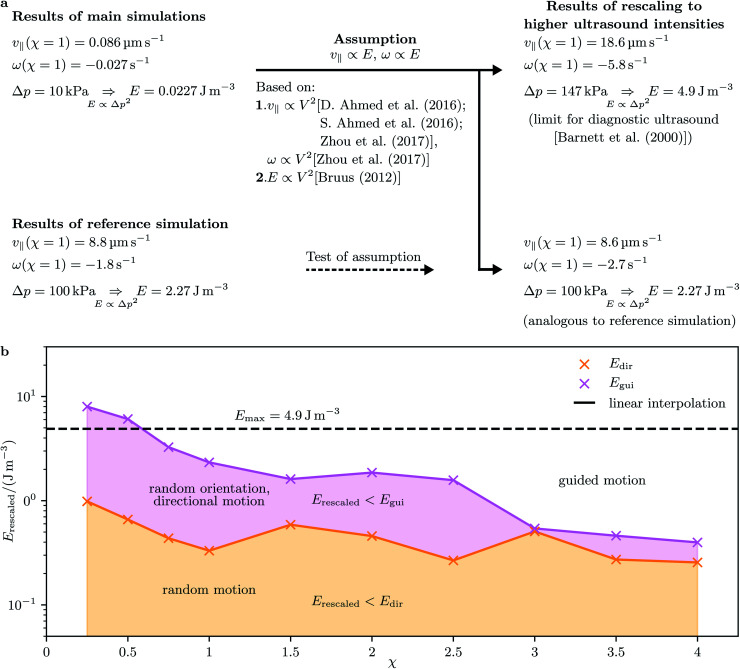
(a) Rescaling of the simulation results to higher values of the pressure amplitude Δ*p* and energy density *E* of the ultrasound. The results from previous studies suggest a quadratic dependence of the translational propulsion velocity *v*_∥_ and angular propulsion velocity *ω* on the amplitude *V* of the applied voltage. Also, the energy density *E* is proportional to *V*^2^. Therefore, the scaling *v*_∥_∝ *E* and *ω* ∝ *E* is assumed. A reference simulation for aspect ratio *χ* = 1, a 10 times increased Δ*p*, and thus a 100 times increased *E* was performed to confirm the assumed scaling. (b) The limiting energy densities *E*_dir_ for directional motion and *E*_gui_ for guided motion (*i.e.*, aligned particle orientation) are shown as functions of *χ*, based on the assumed scaling behavior from (a). The upper limit *E*_max_ for harmless ultrasound in diagnostic applications in human bodies is also shown.

According to amount, the largest velocity *v*_∥_ = −0.098 μm s^−1^, found here for *χ* = 2.5, is about 80% larger than the velocity of cone-shaped particles with aspect ratio *χ* = 0.5 studied in previous work.^51^ Since the energy density *E* = 0.0227 J m^−3^ used in the present work is strongly smaller than the largest energy density *E*_max_ = 4.9 J m^−3^ allowed by the U.S. Food and Drug Administration for diagnostic applications in the human body,^74^ the results can be extrapolated to higher energies. To be able to do this, we need to make some assumption. In experiments, the velocity^39,40,55^ and energy density^75^ were measured to be proportional to the squared amplitude of the driving voltage. The same dependence on the amplitude of the driving voltage was observed in experiments for the angular velocity.^55^ Therefore, we assume that the velocities are proportional to the energy density. To confirm this assumption by direct simulations, we performed an additional simulation for *χ* = 1 and a 10 times larger pressure amplitude, *i.e.*, a 100 times larger energy density, which means an energy density of the same magnitude as *E*_max_. This reference simulation yielded an increase of the translational velocity by a factor ≈103 and an increase of the angular velocity by a factor ≈66, which is in sufficient agreement with the assumed scaling of the velocities with the pressure amplitude. See Fig. 3a for a sketch illustrating the rescaling procedure. Rescaling the highest magnitude of the translational velocity, which is attained at *χ* = 2.5, to the higher energy density *E*_max_ based on the assumed scaling, results in the *E*_max_/*E* = 216 times higher value *v*_rescaled_ = 21.2 μm s^−1^. For *χ* = 2.5, the values of the parameters describing the particle size are *σ* = 0.45 μm and *h* = 1.12 μm. With the higher energy density *E*_max_, the particle would thus move with a speed of roughly 19 body lengths per second. For the largest positive velocity, which corresponds to *χ* = 1, the rescaled speed has the value *v*_rescaled_ = 18.6 μm s^−1^. Since the size parameters are now *σ* = 0.71 μm and *h* = 0.71 μm, this speed equals 26.2 body lengths per second. Depending on the particular application, one can therefore choose an aspect ratio of *χ* = 2.5 or *χ* = 1 to reach a maximal absolute speed or a maximal speed related to the particle length, respectively. For medical applications, compact particles in a certain size range are necessary^6,81,82^ such that they do not block the blood flow. Therefore, the aspect ratio *χ* = 1 could be preferable for these types of applications.

The dependence of *F*_∥,*p*_ and *F*_∥,*v*_ on the aspect ratio is simpler. Both values keep their sign with *F*_∥,*p*_ as a negative force and *F*_∥,*v*_ as a positive force. The magnitude of both values increases until *χ* = 1 fast to *F*_∥,*p*_ = −10.56 fN and *F*_∥,*v*_ = 11.32 fN. Afterwards, the magnitude oscillates a little bit but with the tendency to increase slowly towards *F*_∥,*p*_ = −13.60 fN and *F*_∥,*v*_ = 12.87 fN at *χ* = 4.

We now consider the propulsion parallel to the direction of propagation of the ultrasound wave. The perpendicular propulsion force *F*_⊥_ increases for increasing *χ* slowly until *χ* = 1.5 to *F*_⊥_ = 0.25 fN. Then it is constant until *χ* = 2.5. From *χ* = 2.5 to *χ* = 3 a strong increase occurs to *F*_⊥_ = 0.41 fN. Subsequently, a slow further increase follows. The perpendicular velocity *v*_⊥_ can be seen as roughly constant with *v*_⊥_ = 0.023 μm s^−1^ until *χ* = 2.5 and then it has a small rise to *χ* = 3 with *v*_⊥_ = 0.03 μm s^−1^. Afterwards, it is roughly constant again. This behavior can be understood as follows: in the direction of ultrasound propagation, two opposing forces act on a particle. These are the acoustic radiation force and the acoustic streaming force. Typically, for a particle with a size of about a micrometer, the acoustic radiation force is the dominant one.^83–85^ The scaling behavior of the acoustic radiation force is nontrivial for nonspherical shapes, but for a sphere it scales linearly with the particle volume.^75^ Since we kept the particle volume constant, it is therefore reasonable that the velocity *v*_⊥_ shows no strong change when the aspect ratio of the particle is varied.

The value of the pressure component *F*_⊥,*p*_ of the force *F*_⊥_ increases for increasing *χ* and the value of the viscous component *F*_⊥,*v*_ decreases roughly until *χ* = 2.5, except for a slight intermediate growth of *F*_⊥,*v*_ near *χ* = 1.5. At *χ* = 3, there is a downwards oriented peak in the amplitude for both components. Afterwards, the amplitude increases again for both components, and from *χ* = 3.5 onwards the values of both components increase slightly.

When the particle, which we assume here to be made of gold, is exposed to gravity, the additional force

13 *F*_g_ = *V*_p_(*ρ*_p_ − *ρ*_0_)*g* = 2.29 fN

is exerted on it. Here, *V*_p_ = 0.25 μm^3^ is the volume of the particle, *ρ*_p_ = 1932 kg m^−3^ is its mass density, and *g* = 9.81 ms^−2^ is the constant of gravitation. This force corresponds to a sedimentation velocity 
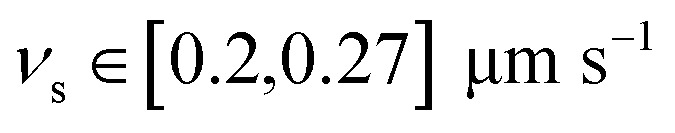
, which follows from the Stokes law (4). As one can see, the influence of gravity on the motion of the particle would not be negligible but much smaller than the propulsion speed that is achieved for suitable values of the aspect ratio *χ* and for the energy density *E*_max_.

Using eqn (7), we can estimate the effect of Brownian motion on the motion of the particle. If we address translational Brownian motion along the orientation of the particle, the diffusion coefficient 
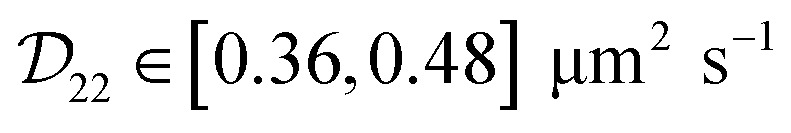
 is relevant. Translational Brownian motion in this direction would therefore be of the order 1 μm s^−1^. This shows that Brownian motion would be visible in experiments but could be clearly dominated by the acoustic propulsion for suitable values of *χ* and *E* = *E*_max_.

Since the time period of sound *τ* = 1 μs is very small and the flow velocity amplitude Δ*v* = 6.75 mm s^−1^ is rather large, both sedimentation and Brownian motion would change the position of a particle only negligibly during a sound period. Therefore, we can expect that sedimentation and Brownian motion cannot affect the acoustic propulsion mechanism. Instead, they lead to independent contributions to the particle motion that can be described by additive terms. This justifies the assumption of a fixed particle in our simulations (see Methods).

The torque acting on the considered particle shapes is for all aspect ratios, according to amount, rather small. It decreases for increasing *χ* from *T* = −0.005 fN μm to *T* = −0.43 fN μm, where the curve has a small local maximum at *χ* = 2. The corresponding angular velocity decreases from *ω* = −0.004 s^−1^ at *χ* = 0.25 to *ω* = −0.033 s^−1^ at *χ* = 1.5, increases afterwards to *ω* = −0.023 s^−1^ at *χ* = 2.5, decreases again to *ω* = −0.06 s^−1^ at *χ* = 3, and remains there for larger values of *χ*.

We now consider the effect of rotational Brownian motion on the particle motion and how it depends on the aspect ratio *χ* of the particle and the energy density *E* of the ultrasound. Note that the acoustic propulsion of the particles depends on *χ* and *E*, whereas Brownian motion depends only on *χ* as long as *E* remains moderate. For energy densities below *E*_max_, we can expect that there is no significant heating of water by the ultrasound so that Brownian motion is not affected. First, we study the occurrence of directional motion by the propulsion. In this case, the motion of the particle must have a preferred direction that originates from the acoustic propulsion. For this, the Brownian rotation of the particle must be very small during a time period *τ* of the ultrasound or, equivalently, *τ* must be small compared to the Brownian reorientation time *τ*_R_ = *D*_R_^−1^ with the particle's rotational diffusion coefficient 
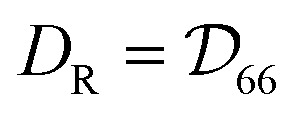
:14
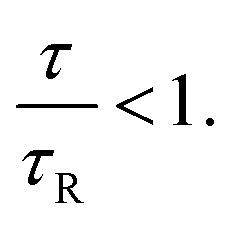


Since *τ* = 1 μs and *τ*_R_ ∈ [0.5,1.6]s for our setup, the first condition is fulfilled for all considered values of *χ* and *E*. Furthermore, the particle must move significantly by its propulsion during the reorientation time *τ*_R_. This means that the persistence length 
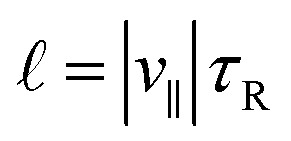
 must be larger than the particle size:15
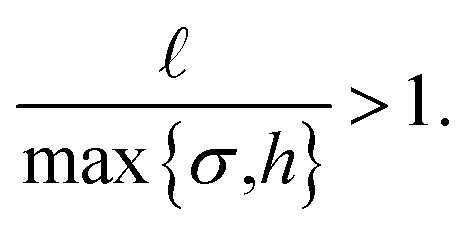


Assuming the scaling |*v*_∥_| ∝ *E*, this condition is fulfilled for energies above16
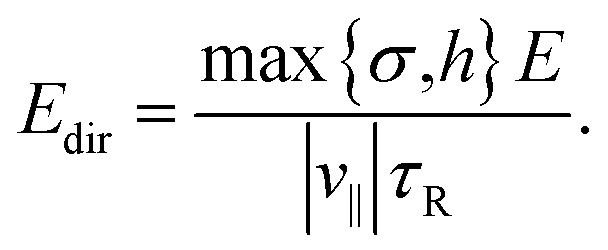


This condition is fulfilled, *e.g.*, if we consider particles with aspect ratio *χ* = 1, where *σ* = *h* = 0.71 μm, *D*_R_ = 1.78 s^−1^, and *τ*_R_ = 0.56 s apply and where we found a speed of 0.1211 body lengths per second, and increase *E* by a factor 15. For *E* < *E*_dir_, one will observe no clear directional motion but random motion. Second, we study the occurrence of guided motion. This occurs when the torque *T* exerted on the particle has a larger effect on its orientation than Brownian rotation has. The condition for guided motion can be written as17
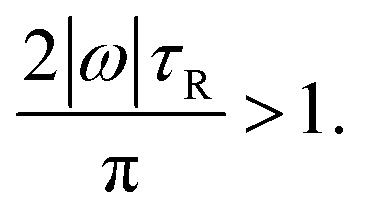


This condition follows from comparing the time π/(2|*ω*|), in which the angular velocity *ω* rotates the particle by 90 degrees, with the Brownian reorientation time *τ*_R_. Assuming the scaling |*ω*| ∝ *E*, this condition is fulfilled for energies above18
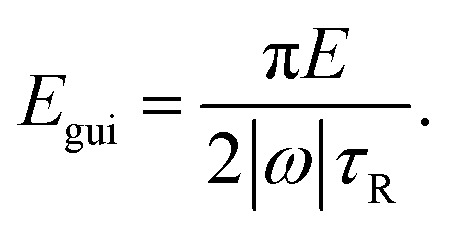


For the values of *χ* and *E* used in our main simulations, the condition (17) is not fulfilled since the small values of the torques that we obtained by our simulations are negligible compared to the rotational Brownian motion of the particles. This indicates that there is either no preferred orientation of the particles or a stable orientation is close to the particle orientation considered in the present work. Considering the aspect ratio *χ* = 4, where we found, according to the amount, the largest torque *T* = −0.43 fN μm and angular velocity *ω* = −0.06 s^−1^, a change of the particle orientation by 90 degrees takes roughly 26 s. On the other hand, the rotational diffusion coefficient *D*_R_ = 0.68 s^−1^ of the particle shows that the particle orientation changes significantly by Brownian rotation on the time scale *τ*_R_ = *D*_R_^−1^ = 1.46 s. This estimate clearly shows that the torques resulting from the ultrasound are so weak that they are dominated by Brownian rotation. However, if the angular velocity is rescaled to the energy density *E*_max_, a change of the particle orientation by 90 degrees needs only 0.12 s and is thus dominant compared to the Brownian rotation. For *E* < *E*_gui_, one will observe no guided motion, but random orientations of the particles that result from Brownian rotation and are typical also for active Brownian particles.^86^Fig. 3b gives an overview about the type of motion of the particles that can be expected for the various considered aspect ratios *χ* and higher energy densities *E*_rescaled_ > *E*. As one can see, *E*_gui_ is larger than *E*_dir_ for all considered values of *χ*.

Concerning the components of the torque, the pressure component *T*_*p*_ decreases (with superimposed fluctuations) for increasing *χ* from *T*_*p*_ = 0.02 fN μm to *T*_*p*_ = −0.36 fN μm, whereas the viscous component *T*_*v*_ fluctuates (with stronger amplitude than for *T*_*p*_) around zero.

In summary, the force and velocity perpendicular to the direction of propagation of the ultrasound wave have a sign change at a particular value of the aspect ratio of the particle, the force and velocity parallel to the propagation direction do not change sign, and the torque is very weak.

**Fig. 4:**
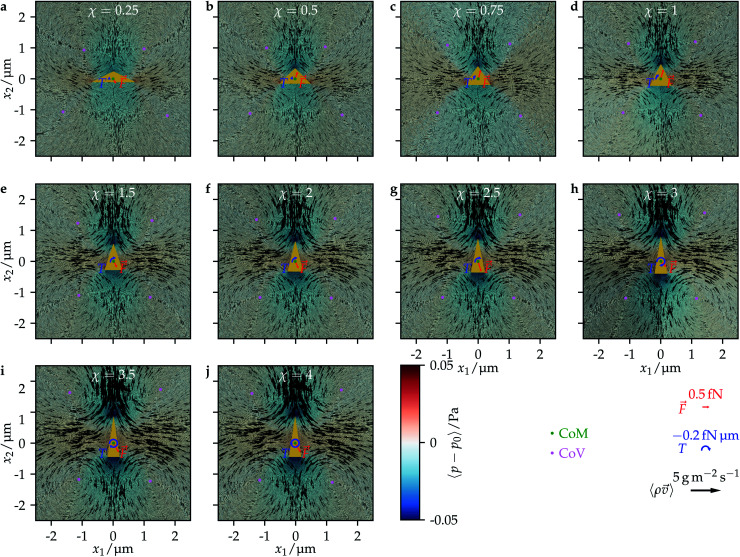
Time-averaged mass-current density 
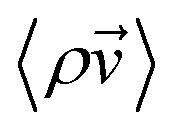
 and reduced pressure 〈*p* − *p*_0_〉 as well as the propulsion force *F⃑* and the propulsion torque *T* acting on a particle for all considered aspect ratios *χ*. The center of mass (CoM) of a particle is marked by a green dot, and the center of a vortex (CoV) is represented by a violet dot.

**Fig. 5:**
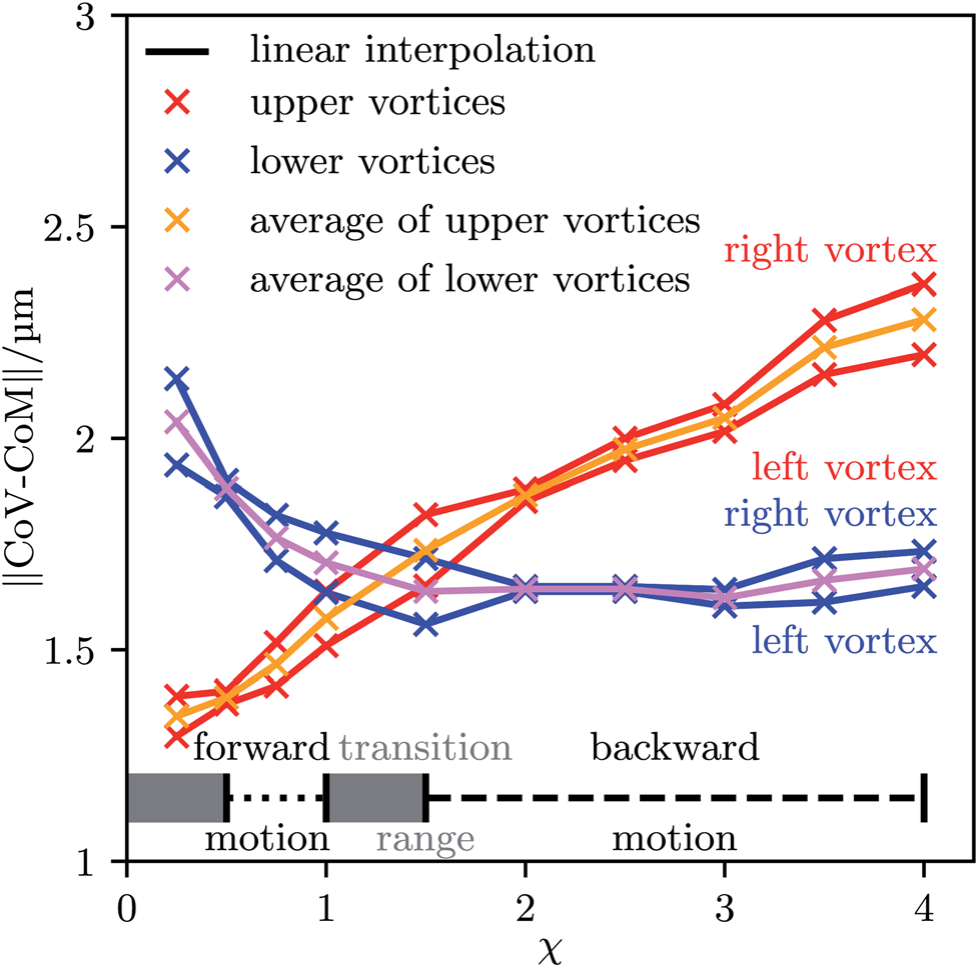
The distance ∥CoV − CoM∥ of the center of a vortex (CoV) from the particle's center of mass (CoM) for each vortex and aspect ratio *χ*. In addition, the average distances of the upper and lower vortices are shown, and the intervals in which our simulations yielded forward or backward motion of the particles are indicated.

Our results are in line with the available theoretical results on ultrasound-propelled particles from the literature. In the theory of Collis *et al.*,^63^ the propulsion direction depends strongly on the acoustic Reynolds number *β* = *ρ*_0_*σ*^2^π*f*/(2*ν*_s_), which ranges for our work between *β* = 0.2 for *χ* = 4 and *β* = 3.1 for *χ* = 0.25. They found that particles can change their propulsion direction up to two times when increasing the acoustic Reynolds number, which is exactly what happens here. According to their theory, this should happen for *β* ∼ *O*(1), which is perfectly in line with the interval of values for *β* we investigated. A similar result was found experimentally.^38,39^ There, long cylinders with spherical caps at the ends corresponding to an aspect ratio *χ* = 4.3 to *χ* = 17.3 are moving towards their concave end,^39^ whereas the short half-sphere cups with aspect ratio *χ* = 0.5 have a propulsion in the opposite direction.^38^ This is qualitatively the same behavior as for our particles, where the long ones with *χ* ≳ 1.5 are moving in the direction opposite to their convex end and the shorter ones with 0.5 ≲ *χ* ≲ 1 are moving towards the convex end. As a qualitative physical picture for the occurrence of the sign change of the propulsion direction, Collis *et al.*^63^ mentioned the *χ*-dependent distances of the vortices that are generated around a particle. Depending on the value of *χ*, either the vortices in front of a particle are closer to the particle than the vortices behind it, or *vice versa*. To study this consideration in the context of our work, we plotted the time-averaged flow field around a particle for each particle shape we addressed. The results are shown in Fig. 4. As one can see, the strength of the vortices increases with the particle's aspect ratio *χ* and also the positions of the vortices relative to the particle change with *χ*. There are two vortices above and below the particle. For low values of *χ*, the vortices in front of the particle are closer to each other and to the center of mass of the particle than the vortices behind it. The opposite applies for large values of *χ*. For some value 1 < *χ* < 1.5, both pairs of vortices have an equal distance from the center of mass. The distances of the vortices from the center of mass are plotted in Fig. 5 as functions of the aspect ratio *χ*. There, also the parameter ranges for which we found forward or backward motion of the particles are indicated. One can clearly see that the direction of propulsion correlates with the relative positions of the vortices. This is reasonable since the vortices lead to a suction at the particle so that those vortices that are closer to the particle determine in which direction it moves.

## IV. Conclusions

We have studied the acoustic propulsion of nano- and microcones powered by a traveling ultrasound wave through direct numerical simulations. Our results show that the propulsion of the particles depends sensitively on their aspect ratio and includes both fast forward and fast backward motion. We found that the direction of motion depends on the distances of the vortices, which are generated around a particle, from the particle. These distances depend on the aspect ratio of the particle, and the particle typically moves towards the vortices that are next to it. The strong dependence of the propulsion on the aspect ratio could be used to separate and sort artificial and natural cone-shaped particles (such as carbon nanocones) in an efficient and easy way with respect to their aspect ratio. For later applications of cone-shaped ultrasound-propelled particles, *e.g.*, in medicine, an aspect ratio of *χ* = 1 was identified as a very suitable choice, since it combines a compact particle shape with a large body-lengths-per-time speed. This finding also suggests to use cone-shaped particles with this aspect ratio as a more efficient particle design for future experiments.

The maximum particle speed of 21.2 μm s^−1^, which we obtained for the still harmless energy density *E*_max_, is of the same order of magnitude as the flow velocity 100 μm s^−1^ (ref. 7) that is typical for blood flow in the capillaries, which constitute by far the largest part of a human's vascular system. However, for a medical application where ultrasound-propelled particles shall transport drugs through the vascular system, this speed is a little too low. It is likely that by optimizing other parameters besides the particle shape, such as the frequency of the ultrasound, the particle speed can further be increased so that the particles can overcome blood flow for harmless ultrasound intensities. Furthermore, the studied particles can already be used in medicine when they are applied outside of the vascular system. An example is an application in the eye, where they could be used to transport drugs through the flow-free vitreous body to the retina.^87^

The obtained results are in good agreement with the literature and expand the understanding of acoustically propelled colloidal particles, which is helpful with regard to future experiments and applications in nanomedicine or materials science. Furthermore, the knowledge about the particle propulsion can be used to model this propulsion when describing the dynamics of the particles *via* Langevin equations^88,89^ or field theories based on symmetry-based modeling,^90,91^ the interaction–expansion method,^92,93^ classical dynamical density functional theory,^94,95^ or other analytical approaches on time scales that are much larger than the period of the ultrasound.

In the future, this study should be extended by considering other particle orientations and studying how the propulsion depends on the angle between the particle orientation and the direction of propagation of the ultrasound. Furthermore, the dependence of the propulsion on parameters like the ultrasound frequency, pressure amplitude, and fluid viscosity still need to be investigated. For the latter case, the results of previous experiments suggest that the propulsion speed increases for decreasing viscosity.^27,29,30,46,49^ It is, therefore, likely that the ratio of acoustic propulsion and Brownian motion depends on the viscosity.

## Conflicts of interest

There are no conflicts of interest to declare.

## Supplementary Material
